# Quantitative difference of oral pathogen between individuals with gastric cancer and individuals without cancer

**DOI:** 10.18632/oncotarget.28034

**Published:** 2021-08-17

**Authors:** Gyselle Ribeiro de Carvalho Oliveira, Carla de Castro Sant’ Anna, Letícia Martins Lamarão, Adriana Costa Guimarães, Carlos Machado da Rocha, Marcelo de Oliveira Bahia, Carolina Rosal de Souza, Danielle Queiroz Calcagno, Paulo Pimentel de Assumpção, Rommel Rodriguez Burbano

**Affiliations:** ^1^School of Dentistry, Federal University of Pará, Belém, Pará, Brazil; ^2^High Complexity Oncology Unit, Federal University of Pará, Rua dos Mundurucus, Hospital Universitário João de Barros Barreto, 2º piso da UNACOM, Belém, Pará, Brazil; ^3^Ophir Loyola Hospital, Av. Gov Magalhães Barata, Belem, Pará, Brazil; ^4^Foundation Center for Hemotherapy and Hematology, Travessa Padre Eutíquio, Belém, Pará, Brazil; ^5^Federal Institute of Pará, Belém, Pará, Brazil; ^6^Institute of Biological Sciences, Federal University of Pará, Belém, Pará, Brazil

**Keywords:** periodontal disease, microbiome, gastric cancer, oral pathogens

## Abstract

The loss of teeth and lack of oral hygiene have been associated with the risk of developing gastric cancer (GC) in several populations evidenced in epidemiological studies. In this study, we quantitatively compared the proportion of oral pathogens in individuals with gastric cancer and individuals without cancer in a referral hospital in the city of Belém, Brazil. This study evaluated 192 patients with GC and 192 patients without cancer. Periodontal clinical examination was performed, and all individuals were submitted to the collection of salivary and dental biofilms. When comparing the median periodontal indexes in the gastric and cancer-free groups, it was statistically significant (*p* < 0.001) in the gastric cancer group compared to the probing depth of the periodontal pocket. Levels of bacterial DNA were observed in saliva and dental plaque, with a statistically significant difference (*p* < 0.001) between individuals with cancer and without neoplasia in all the bacteria surveyed. Significant relationships (*p* < 0.001) between biological agents and GC have been found in bacterial species that cause high rates of periodontal pathology and caries. The results suggest a different quantitative association in the presence of oral pathogens between individuals without cancer and patients with GC. As noted, it cannot be said that the bacteria present in the oral cavity increase the risk of gastric cancer or are aggravating factors of the disease. However, it is worth mentioning that, as it is part of the digestive system, the lack of care for the oral cavity can negatively affect the treatment of patients with gastric cancer.

## INTRODUCTION

Sporadic gastric cancer is the result of several molecular changes induced by environmental factors, including admission of diets with high salt levels (mainly with high sodium concentrations); poor food preservation; increased N-nitrous compounds in the gastric mucosa; antioxidant/vitamin deficiencies (e.g., vitamin C); *Helicobacter pylori* infection; proinflammatory cytokine gene polymorphism; and prolonged consumption of alcohol and tobacco [1, 2]. The cumulative effect of these aggressions on the gastric epithelium over the years leads to the development of neoplasia, so gastric cancer usually has a high incidence in the sixth decade of life in individuals with chronic atrophic gastritis and intestinal metaplasia [3]. Cancer is one of the major public health problems worldwide, and an estimated 19.3 million new cancer cases occurred in 2020 [4]. Among the different cancers that affect humans, gastric cancer (ICD-10 C16) is the third leading cause of cancer-related deaths in the world and the fifth most frequent malignant tumor [5].

In the northern region of Brazil, the state of Pará has mortality rates historically above the Brazilian average [6]. Gastric cancer has intrinsic resistance to radiotherapy and chemotherapy, and prevention is probably the most effective means of reducing mortality caused by this neoplasm [7]. Currently, despite some advances in the treatment of gastric neoplasia, surgery is still the main curative treatment [8, 9]. These facts highlight the severity of this pathology and the need to develop new studies that may help in identifying the peculiar genetic characteristics of a tumor, increasing the ability to predict the behavior of this tumor, and allowing the establishment of more precise therapeutic approaches [10].

The human oral cavity is home to one of the most diverse microbiotas in the human body [11, 12]. Bacterial cells are responsible for the two most common diseases in humans: tooth decay and periodontal disease. Human health is also influenced by oral hygiene, where pathologies of the mouth have been linked to various diseases, such as diabetes mellitus, chronic obstructive pulmonary disease, cardiovascular disease, endocarditis, and the presence of bacteria in the blood [13]. The human mouth is part of the gastrointestinal system, and oral health is related to the gastrointestinal carcinogenic process [14]. Currently, the etiology of dental caries is considered an infectious disease caused by specific bacteria in which a mixed bacterial-ecological interaction is responsible for the appearance of the pathogenic lesion [15].

The imbalance of the oral microbiota is related to several chronic pathologies associated with gastrointestinal diseases. In addition, scientific advances have shown the correlation between periodontal diseases and the development of gastric cancer [16]. The risk of the appearance of precancerous gastric lesions increases in the presence of virulence of periodontal factors, in addition to the diversity of the oral microbial environment [17].

Periodontal pathogens are receiving growing interest in elucidating the etiology of cancer. The pathogenesis of periodontitis is related to important functions of periodontal bacteria, causing destruction of dental support tissue, which can lead to tooth loss [18]. Thus, the association of periodontal pathogens and pancreatic and oral cancer has been observed in epidemiological research (Table 1) [19–23].

**Table 1:** Main bacteria involved in oral pathogenesis studied in patients with gastric cancer

Bacterial species	Disease location
*Streptococcus sobrinus* (Sb)	dental cavity
*Porphyromonas gingivalis* (Pg)	periodontal disease
*Treponema denticola* (Td)	periodontal disease
*Aggregatibacter actinomycetemcomitans* (Aa)	periodontal disease
*Tannerella forsythia* (Tf)	periodontal disease
*Streptococcus mutans* (Sm)	dental cavity

The invasion of oral bacteria in the human body and its products, including inflammatory molecules, can occur in two main ways: through the bloodstream or through the digestive tract [24]. To check the association between bacteria in the oral cavity and gastric cancer, this study aimed to quantify and compare between patients with gastric cancer and cancer-free (CF) patients and the bacteria responsible for periodontal disease, including *Aggregatibacter actinomycetemcomitans* (*A. actinomycetemcomitans*), *Treponema denticola* (*T. denticola*), *Porphyromonas gingivalis* (*P. gingivalis*) and *Tannerella forsythia* (*T. forsythia*), in addition to quantifying and comparing between patients from gastric cancer and CF groups in relation to the bacteria responsible for the dental caries process, such as *Streptococcus mutans* (*S. mutans*) and *Streptococcus sobrinus* (S. *sobrinus*), and to quantify and compare the CF group with the stages of gastric cancer.

## RESULTS

When comparing the distribution of the participants’ characteristics by the status of gastric cancerous lesions, the participants were mostly brown (77.34%) and male (61.98%) and had an average age of 52 years. There was a higher prevalence of nonsmokers in the groups (*p* = 0.06). A higher average body mass index (BMI) was significantly lower in the group with gastric cancer than in the FC group (*p* < 0.001), as well as when comparing the average number of bleeding sites and the average PPD (*p* < 0.001), but there was no statistically significant difference when we made the same comparison of sites with CAL (*p* = 0.1040) (Table 2).

**Table 2:** Distribution of participant characteristics by gastric cancerous lesion status

Characteristics of the participants	Gastric Lesion		
	GC	CF	*p*-value^1^
**Sex (%)**			
Men	61.98	61.98	1^1^
Women	38.02	38.02	
**Age, years**			
Mean (SD)	52.57 (10.916)	51.20 (13.235)	0.2707^2^
**Education (%)**			
Less than high school	65.62	46.87	
High school	23.96	36.98	0.0281^1^
Some college or graduate	10.42	16.15	
**Race (%)**			
Black	5.21	1.56	
Brown	68.75	85.94	
Yellow	3.12	2.08	0.0670^1^
White	18.23	8.34	
Amerindian	4.69	2.08	
**BMI (kg/m^2^)**			
Mean (SD)	23.46 (2.787)	25.25 (2.173)	0.001^2^
**Smoking Status**			
Positive	32.29	19.79	
Negative	67.71	80.21	0.0639^1^
**Alcohol Consumption (%)**			
Positive	32.29	19.79	
Negative	67.71	80.21	0.0639^1^
**PCR urease (%)**			
Positive	87.50	82.81	
Negative	12.50	17.19	0.4630^1^
**cagA (%)**			
Positive	67.71	55.73	
Negative	32.29	44.27	0.1102^1^
**EBV (%)**			
Positive	18.23	3.65	
Negative	81.77	96.35	0.0021^1^
**HPV (%)**			
Positive (HPV16)	2.08	0	
Positive (HPV18)	2.08	0	0.1195^1^
Negative	95.84	100	
**Bleeding sites (%)**			
Mean (SD)	30.58 (4.157)	22.42 (6.990)	0.001^2^
**Sites with PPD ≥ 3 mm (%)**			
Mean (SD)	20.59 (3.879)	18.35 (6.468)	0.001^2^
**Sites with CAL ≥ 3 mm (%)**			
Mean (SD)	32.01 (4.264)	31.06 (6.7413)	0.1040^2^

Abbreviations: BMI, Body Mass Index; GC, Gastric Cancer; CF, Cancer Free; PPD, Pocket Probing Depth; CAL, Clinical Attachment Level. ^1^ *p*-value by chi-square test. ^2^ *p*-value for *t*-test.

Regarding the levels of bacterial DNA, it was observed that in saliva, there were statistically significant differences between the gastric cancer and CF groups in all bacteria surveyed; however, in dental plaque, these differences were statistically significant only in *P. gingivalis* and *T. forsythia* (Table 3).

**Table 3:** Comparison of median bacterial DNA levels in relation to gastric cancerous lesions

Bacterial DNA levels	Median (IQR)^1^		
	GC	FC	*p*-value^2^
**Saliva**			
*A. actinomycetemcomitans* (*Aa*)	2.3594 (2.1367–2.5992)	0.9280 (0.8140–1.1065)	<0.001
*P. gingivalis* (*Pg*)	1.3808 (1.2485–1.6380)	2.0568 (1.8392–2.2436)	<0.001
*T. denticola* (*Td*)	3.6482 (3.2499–4.2853)	19.0366 (17.7566–19.5566)	<0.001
*T. forsythia* (*Tf*)	2.0492 (1.9708–2.1451)	7.0225 (6.7302–7.3446)	<0.001
*S. sobrinus* (*Sb*)	3.2498 (3.0548–3.4387)	0.5314 (0.5127–0.5517)	<0.001
*S. mutans* (*Sm*)	5.1746 (4.8102–5.4868)	9.4285 (9.2324–9.6245)	<0.001
**Dental Plaque**			
*A. actinomycetemcomitans* (*Aa*)	1.7214 (1.6410–1.8276)	1.5863 (1.4225–1.6838)	<0.001
*P. gingivalis* (*Pg*)	6.5364 (5.4225–7.3187)	10.5563 (8.7321–11.1147)	<0.001
*T. denticola* (*Td*)	1.5241 (1.4495–1.5873)	1.8279 (1.7559–1.9116)	<0.001
*T. forsythia* (*Tf*)	3.5175 (3.2131–3.9476)	6.2397 (5.8068–6.8193)	<0.001

Abbreviations: GC, Gastric Cancer; CF, Cancer Free; IQR, interquartile range. ^1^Medians of absolute bacterial counts (ng/ml) adjusted for age. ^2^ *p*-value for Mann-Whitney test.

Comparing the median bacterial DNA between CF individuals and the stages of gastric cancer lesions, it was observed that in the saliva, the median of all bacteria showed statistically significant differences in all stages. On the plaque, the same happened to the group of patients with gastric cancer, and all bacteria had different median values at all stages when compared to the group of individual CFs. We also observed that the largest number of patients had stage IV disease, which is consistent with the lack of oral care and prevention of the gastrointestinal tract (Table 4).

**Table 4:** Adjusted median bacterial DNA levels between non-cases and the stages of gastric cancer lesions

Median (*p*-value for Mann-Whitney test)									
	Non-case	IA (*n* = 3)	IB (*n* = 9)	IIA (*n* = 11)	IIB (*n* = 7)	IIIA (*n* = 29)	IIIB (*n* = 19)	IIIC (*n* = 6)	IV (*n* = 108)
**Saliva**									
*A. actinomycetemcomitans* (*Aa*)	0.9280	2.6027 (<0.001)	2.0648 (<0.001)	2.2591 (<0.001)	2.3052 (<0.001)	2.2635 (<0.001)	2.2482 (<0.001)	2.4598 (<0.001)	2.4425 (<0.001)
*P. gingivalis* (*Pg*)	2.0568	1.5840 (0.0061)	1.3652 (<0.001)	1.3757 (<0.001)	1.2080 (<0.001)	1.3358 (<0.001)	1.3113 (<0.001)	1.3711 (0.001)	1.4472 (<0.001)
*T. denticola* (*Td*)	19.0366	4.4287 (<0.001)	3.8353 (<0.001)	3.5581 (<0.001)	3.7817 (<0.001)	4.0994 (< 0.001)	3.6386 (<0.001)	3.1388 (<0.001)	3.6437 (<0.001)
*T. forsythia* (*Tf*)	7.0225	2.1872 (<0.001)	1.9866 (<0.001)	2.0532 (<0.001)	2.0947 (<0.001)	1.9911 (< 0.001)	2.0538 (<0.001)	1.9860 (<0.001)	2.0551 (<0.001)
*S. sobrinus* (*Sb*)	0.5314	3.0582 (<0.001)	3.1276 (<0.001)	3.4207 (<0.001)	3.0464 (<0.001)	3.0885 (<0.001)	3.4207 (<0.001)	3.0265 (<0.001)	3.2912 (<0.001)
*S. mutans* (*Sm*)	9.4285	4.9918 (<0.001)	5.0705 (<0.001)	5.1590 (<0.001)	5.2186 (<0.001)	5.0729 (<0.001)	5.0771 (<0.001)	5.6576 (<0.001)	5.2480 (<0.001)
**Plaque**									
*A. actinomycetemcomitans* (*Aa*)	1.5863	1.8021 (0.0150)	1.8276 (0.0011)	1.7256 (0.0013)	1.8058 (0.0014)	1.7469 (<0.001)	1.6996 (<0.001)	1.7489 (0.0068)	1.7056 (<0.001)
*P. gingivalis* (*Pg*)	10.5563	8.0431 (<0.001)	6.1636 (<0.001)	6.5555 (<0.001)	7.2602 (<0.001)	6.8336 (<0.001)	5.7006 (<0.001)	7.2513 (<0.001)	6.5161 (<0.001)
*T. denticola* (*Td*)	1.8279	1.5064 (0.0037)	1.5586 (<0.001)	1.4896 (<0.001)	1.4279 (<0.001)	1.4814 (<0.001)	1.5077 (<0.001)	1.4538 (<0.001)	1.5074 (<0.001)
*T. forsythia* (*Tf*)	6.2397	3.0745 (<0.001)	3.3550 (<0.001)	3.2835 (<0.001)	3.2500 (<0.001)	3.5000 (<0.001)	3.6156 (<0.001)	3.4892 (<0.001)	3.5950 (<0.001)

*n* = number of patients with gastric cancer in each stage.

For bacteria related to periodontal disease, the DNA levels of the bacteria studied here were significantly associated with gastric cancer. In the plaque, this association was also found in all bacteria analyzed (Table 3). In the subgingival plaque, the levels of microorganisms can be less diluted than in saliva and, therefore, the most sensitive qPCR in their detection.

## DISCUSSION

An etiological factor of gastric cancer is the presence of the bacterium *H. pylori*, which can be considered statistically a confounding factor, but the analysis shows that there is no difference between the two groups. It is difficult to find a group free of *H. pylori* bacteria in northern Brazil, since it is a public health problem [25]. HPV virus has a controversial role in gastric carcinogenesis, and there was no difference between the groups with gastric cancer and CF. On the other hand, EBV virus is a virus capable of inducing cancer in a subset of patients. This virus caused a significant difference between individuals with cancer (*p* = 0.0021) and the CF group. In previous works by our research group, this difference was also revealed [25, 26].

Oral health problems and tooth loss had a positive correlation with gastric cancer. We also observed gingival bleeding rates with a higher median in cases with injury than in those without injury, and we did not find other significant differences in rates of periodontal disease. These oral disorders have been previously reported [27].

Coker et al. [28] observed an overrepresentation of *T. forsythia* in gastric cancer compared to other precancerous stages. Other studies have not found an association of *T. forsythia* in patients with precancerous gastric lesions [14, 17]. In our study, *T. forsythia* had a low quantification in individuals with gastric cancer in relation to CF individuals (*p* < 0.001), which is probably a regional phenomenon, as we have not found similar studies in South America.

Ndegwa et al. [29] found no increased risk of gastric cancer for individuals with high levels of dental plaque. However, these authors agree that this is a conflicting result because periodontal disease, characterized by chronic inflammation and microbial dysbiosis, is a significant risk factor for orodigestive carcinogenesis.

One of the fundamental bacteria for the caries process that affects more than 90% of the world population is the species *S. mutans*. However, other species may contribute to the onset of the disease, such as acidogenic and aciduric/acidophilic species [30]. However, *S. mutans* gained attention from the scientific community in the late 1950s and clinical and laboratory research in the 1960s, where its etiological relevance in the process of tooth decay was observed [31, 32].

Our research group decided to include *S. mutans* and *S. sobrinus* in this study with gastric cancer, since these two gram-positive bacteria, associated with dental caries, had no statistical association with patients with precancerous gastric lesions in relation to control subjects. However, it is known that an altered oral microbiota has been associated with the development of periodontal pathogenic bacteria/oral bacteria and oral, esophageal, and pancreatic cancer [21–23]. In the present study, *S. sobrinus* was more frequent in patients with gastric cancer than in CF individuals. On the other hand, there was a lower frequency of *S. mutans* in patients with gastric cancer than in CF individuals. To the best of our knowledge, this is the first time that these associations have been reported in the literature on gastric cancer.

*P. gingivalis* was found in abundance in patients with oral squamous cell carcinoma. Tumorigenesis models indicated a direct relationship between *P. gingivalis* and carcinogenesis [33]. *T. denticola* is also associated with severe periodontitis. This anaerobic spirochete is an invasive bacterium due to its main virulence factor, chymotrypsin-like proteinase. *T. denticola* was detected in samples of orodigestive tumors [18]; however, in our results, this phenomenon was not repeated in gastric cancer, where *P. gingivalis* and *T. denticola* were more abundant in CF individuals (Table 3). The fact that distant organs are not involved is an indication that increases the controversy that these two bacteria may not have systemic tumorigenic effects in addition to the local effects in their native territory, the oral cavity.

We found evidence that the levels of bacteria related to tooth decay are associated with gastric cancer in saliva. However, this has not been reported in precancerous gastric lesions [17].

There are indications that higher levels of periodontal pathogens are related to more aggressive forms of periodontitis [34]. Oral health conditions and bacterial profiles are changeable, and the bacterial risk factors identified for malignancy can have important implications for the prevention of cancerous processes. The characteristics of the oral microbiome in individuals with gastric cancer may allow the realization of a method of tracking gastric cancer through the detection of the oral microbiome. In addition, periodontal microbiota dysbiosis is associated with several chronic diseases. The association and role of P. gingivalis incursion in arterial cardiovascular disease has been demonstrated, for example [35].

The large size of our sample and the clinical information generated by the patients in this study, although only a start, may allow analysis of the oral microbiota to assist gastric cancer therapy and help develop individualized approaches to cancer prevention and treatment stomach. The results suggest a possible systemic disease related to or influenced by periodontal disease.

## MATERIALS AND METHODS

### Patients and samples

This study evaluated 192 patients diagnosed by endoscopy with gastric cancer and 192 patients without cancer, methodologically matched in gender and age, from northern Brazil. This study was conducted in accordance with the Declaration of Helsinki, and all participants signed the Informed Consent Form in advance (CAAE 10272913.8.3001.5550) by the Ethics Committee of João de Barros Barreto University Hospital. Patients diagnosed with gastric cancer had not started treatment, and neither group had received antibiotic therapy in the last 3 months. The patients underwent oral health clinical examination, where saliva and plaque samples for microbiological analysis by molecular biology were collected and received oral hygiene instructions.

### Detection of *H. pylori* and cagA

*H. pylori* produces urease to convert urea to ammonia, and this property is exploited by a rapid urease test (Promedical, Juiz de Fora, Brazil) to detect the presence of *H. pylori* in gastric samples. When urease was detected, the pH and the color of the solution were changed. PCR was used to confirm negative results and detect the presence of the cagA gene in the *H. pylori*-positive (*H. pylori*+) samples. The oligonucleotides used here were described by Covacci et al. [36]. The methodology was carried out according to de Souza et al. [25].

### Detection of EBV

EBV was detected in gastric samples by using a 30-bp biotinylated probe (5′-AGACACCGTCCTCACCACCCGGGACTTGTA-3′) that is complementary to the most abundant viral product in latently infected cells, EBV-encoded small RNA-1 (Eber1) [37]. EBV is known to infect lymphocytes, but these cells were not included in the present analysis. For this assay, RNA *in situ* hybridization (ISH) was used, according to the instructions for Souza et al. [26]. Samples were considered positive when 5% or more of the epithelial cells were stained brown/red.

### Detection of HPV

Nested PCR was used to classify the samples as HPV positive or negative. The first PCR used the generic PGMY09/11 primers described by Gravitt et al. [38], which amplify the L1 region of HPV, and the second PCR used the GP5+/6+ primers described by Jacobs et al. [39]. The PCR products were separated, identified, and genotyped by sequencing the PCR product with the GP5+ primer. All the nucleotide sequences obtained in these analyses were compared with the GenBank/NCBI database using the BLAST alignment search tool (http://blast.ncbi.nlm.nih.gov/Blast.cgi). The E6 and E7 oncoprotein expression of HPV 16 and 18 was investigated by RT-PCR as described by Chang et al. [40].

### Periodontal clinical examination

The assessment was double blind, as the diagnosis of patients involved in oral research was performed by two calibrated dentists.

Periodontal examination was performed using a North Carolina 15 millimeter periodontal (Hu-Friedy^®^, Chicago, USA) probe at six dental sites (mesiobuccal, buccal, distobuccal, distalingual, lingual, and mesioingual) of all teeth present. Clinical attachment level (CAL), defined as the distance from the cementum-enamel junction to the free gingival margin in millimeters and pocket probing depth (PPD), was defined as the distance between the free gingival margin and the most coronal portion of the junctional epithelium (deep groove/pocket). The dichotomous record for each dental surface of the gingival bleeding test was considered positive if it occurred within 15 seconds after the assessment of the probing depth.

### Salivary collection and dental biofilm

All subjects were asked to chew a piece of paraffin and then gently sputter 2 to 5 ml of saliva directly into a refrigerated collection tube.

Six dental biofilm samples were collected from each subject with sterile periodontal curettes: four referring to the first molar supragingival plaque or to the most posterior tooth in each quadrant and two subgingival plaque samples from teeth with deeper periodontal pockets. Plaque samples were immediately transferred to a prelabeled sterile tube containing 200 ml TE buffer. Saliva samples and plates were carefully shaken for 30 seconds, immediately placed in a cool box containing ice and transferred to the laboratory within 1 h for further processing.

### Quantitative analysis of real-time polymerase chain (qPCR) expression

qPCR was performed to detect presence/absence and quantify bacterial DNA in saliva and plaque samples. Bacterial samples were dissolved at 4°C and then washed in 1 ml of 10 mm Tris–HCl (pH 7.5) and 1 mm of EDTA buffer. The total genomic DNA of the bacterial samples was isolated with the MaterPure DNA purification kit (Epicenter, Madison, WI). An additional 10 μl proteinase K (Qiagen, Venlo, NED) solution 10 mg/ml in TES buffer – 10 mm Tris–HCl, pH 8.0; 1 mm EDTA; 100 mm NaCl and 2 μl mutano-lysin (Sigma-Aldrich St. Louis, USA) [5000 U/ml in phosphate-buffered saline buffer (PBS)] were added to lysozyme (Sigma-Aldrich St. Louis, USA) stock solution (100 mg/ml in TES buffer) to ensure the release of DNA from all bacteria in the samples, followed by a phenol/chloroform/isoamyl alcohol extraction procedure. Subsequently, the total genomic DNA concentration of each sample was quantified using a 260 nm and 280 nm UV spectrophotometer (NanoDrop 2000C, ThermoScientific, Wilmington, USA). The final concentration of each DNA sample extracted from saliva was adjusted to 10 ng/ml to be subjected to qPCR methodology. The qPCR reaction mixture contained a total volume of 25 μl, which contained 10 X diluted buffer, 2.5 mM DNTPs/ea, 10 pmole/ml primers, 5 U/ml Taq, 50 mm MgCl2 and 20 ng/ml DNA probe. The 25 μl DNA amplification reaction was composed of a dilution (1X) of QuantiTect SYBR Green PCR Master Mix (Qiagen, Venlo, NED), 10 ~ 100 ng of total genomic DNA, and 0.4 mM of each primer. The species-specific PCR primers used in this study are listed in Table 5.

**Table 5:** Species-specific primers that were utilized for real-time quantitative PCR (qPCR) (Salazar et al., 2013)

Selected oral pathogen	Primer pairs (5′~3′)	bp^a^
**Periodontal disease pathogens**		404 [41]
*P. gingivalis* Gram-negative anaerobe	AGG CAG CTT GCC ATA CTG CG ACT GTT AGC AAC TAC CGA TGT	
*T. forsythensis* Gram-negative anaerobe	TAC AGG GGA ATA AAA TGA GAT ACG TTC ACC GCG GAC TTA ACA GC	250 [42, 43]
*T. denticola* Gram-negative anaerobe	TAA TAC ATG TGC TCA TTT ACA T TCA AAG CAT TCC CTC TTC TTC TTA	316 [41]
*A. actinomycetemcomitans* Gram-negative anaerobe	ATT GGG GTT TAG CCC TGG T GGC ACA AAC CCA TCT CTG A	250 [43]
**Dental caries pathogens**		479 [44]
*S. mutans* Gram-positive anaerobe	TCG CGA AAA AGA TAA ACA AAC A GCC CCT TCA CAG TTG GTT AG	
*S. sobrinus* Gram-positive anaerobe	TTC AAA GCC AAG ACC AAG CTA GT CCA GCC TGA GAT TCA GCT TGT	88 [45]

^a^base pairs of amplicon length.

Serial (10-fold) dilutions of *P. gingivalis* (ATCC33277), *T. forsythensis* (ATCC43037), *T. denticola* (ATCC35404), *A. actinomycetemcomitans* (ATCC29522), *S. mutans* (UA159) and *S. Sobinus* (OMZ176) was used in each reaction as an external control for absolute quantification of bacterial targets. DNA levels for all six bacterial species were measured in saliva samples, and four bacterial species relevant to periodontal disease (*A. actinomycetemcomitans*, *P. gingivalis*, *T. denticola*, *T. forsythia*) were measured in each plaque sample. The colonies of *S. sobrinus* and *S. mutan*s were tested in saliva instead of in plaques because plaque samples were collected from specific locations of teeth showing deeper periodontal pockets and may not truly reflect the overall prevalence of these cariogenic pathogens in saliva.

The use of qPCR methodology involving stimulated total saliva is a scientifically effective methodology for assessing the prevalence of *S. sobrinus* and *S. mutans* [17]. The analytical specificity of the fluorescent signal was determined based on subsequent melting curve analysis. All reactions were performed in duplicate, and the final analysis was based on the average of the two reactions. Data were analyzed using Opticon Monitor 2 software (MJ Research, Waltham, USA).

## CONCLUSIONS

The results found in this study were quite evident, increasing the existing knowledge on the relationship between bacteria in the oral cavity and gastric cancer. As noted, according to the results, it cannot be said that bacteria present in the oral cavity increase the risk of gastric cancer or are aggravating factors of the disease. However, it is worth mentioning that, as it is part of the digestive system, the lack of care for the oral cavity can negatively affect the treatment of patients with gastric cancer.
